# Late Closure of a Stage III Idiopathic Macular Hole after Pars Plana Vitrectomy

**DOI:** 10.4274/tjo.33603

**Published:** 2015-12-05

**Authors:** Filiz Afrashi, Zafer Öztaş, Serhad Nalçacı

**Affiliations:** 1 Ege University Faculty of Medicine, Department of Ophthalmology, İzmir, Turkey

**Keywords:** Macular hole, optical coherence tomography, vitrectomy

## Abstract

A 57-year-old female presented to our hospital with decreased vision in her right eye. Detailed ocular examination was performed, and a macular hole was detected in the right eye. The presence of a full-thickness stage III macular hole was confirmed with optical coherence tomography (OCT) imaging. Pars plana vitrectomy followed by long-acting gas tamponade (C3F8) was performed as treatment. One month after surgery, clinical examination revealed a persistent macular hole, confirmed by an OCT scan. Although the patient was scheduled for reoperation, the surgery was postponed due to personal reasons of the patient. Surprisingly, after five months, a closure pattern with accompanying epiretinal membrane was observed in the macular hole area. The closure of the macular hole was completed without any further intervention 8 months post-surgery. In cases of unclosed macular hole after the first surgery, if a second surgery cannot be performed, follow-up with OCT recommended due to the possibility of spontaneous closure. However, spontaneous closure of a persistent macular hole following PPV is rare, so early diagnosis and surgical repair of unclosed macular holes must remain the primary goal.

## INTRODUCTION

The successful closure of macular holes by pars plana vitrectomy (PPV) was first reported by Kelly and Wendel.^1^ Vitreous surgery, which consists of PPV and removal of adherent cortical vitreous from the surrounding retinal surface followed by gas tamponade, can be used to repair macular holes with postoperative face-down positioning. The removal of the internal limiting membrane (ILM) during macular hole surgery has been shown to improve success rates.^2^

Herein, we report unexpected spontaneous closure of a full-thickness idiopathic macular hole that occurred after a failed vitrectomy surgery. To the best of our knowledge, this is the first report describing the late spontaneous closure of a stage III idiopathic macular hole after a failed surgery.

## CASE REPORT

**Figure 1:**
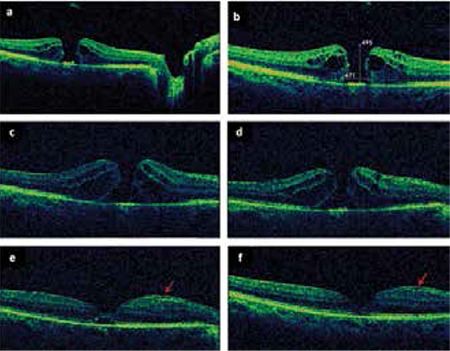
a) Stage III full-thickness macular hole, b) Preoperative base diameter and height of macular hole; Persistent macular hole c) 1 month, d) 3 months, and e) 5 months after surgery. At 5 months, closure pattern in macular hole area with a mild epiretinal membrane (arrows) is visible, f) Spontaneous closure was completed 8 months after surgery

A 57-year-old otherwise healthy female with a one month history of right visual deterioration was evaluated. An ophthalmological examination including slit-lamp biomicroscopy, best corrected visual acuity (BCVA) assessment, intraocular pressure (IOP) measurement, and fundoscopy was performed. Initial BCVA was 20/125 in the right eye and 20/20 in the left eye. Anterior segments were normal in both eyes. Preoperative and postoperative IOP measurements were within normal limits (range, 15 to 21 mmHg). Funduscopic examination revealed a full-thickness macular hole with incomplete posterior vitreous detachment, and optical coherence tomography (OCT) scan showed a stage III full-thickness macular hole with cystic formation and elevated borders (Figure 1a). The base diameter of the hole was 671 microns and the hole depth was 493 microns (Figure 1b).

Standard three-port PPV and gas tamponade (C3F8) procedures were performed to surgically repair the macular hole. The ILM was dissected but not successfully removed due to tight adhesions at the macula. Postoperative follow-up examinations were performed weekly in the first month, and monthly thereafter. At one month after surgery, the intravitreal gas had been resorbed and OCT scan revealed an unclosed macular hole (Figure 1c, 1d). An additional surgery for the unclosed macular hole was proposed but the patient postponed the second surgery due to personal reasons. At 5 months after surgery, OCT showed a closure pattern in the macular hole area with epiretinal membrane (ERM) formation (Figure 1e). The closure was completed 8 months after surgery (Figure 1f). Final BCVA was limited at 20/100 due to cataract progression. The follow-up period was 24 months and there was no reccurence during this period.

## DISCUSSION

The spontaneous closure of idiopathic stage III and IV macular holes is quite a rare event.^3,4,5,6,7,8,9,10^ Therefore, PPV is a required intervention in cases with stage III and IV macular holes. However, the incidence of persistent macular holes after PPV is between 4% and 13%.^11,12,13^ Early reoperation is a standard approach in the current management of these persistent macular holes, which have a poor prognosis.^14^ The present study describes the late spontaneous closure of a persistent idiopathic stage III full-thickness macular hole after vitrectomy surgery.

Fibrocellular proliferation is one of the mechanisms most suspected in the persistence of macular holes or the reopening of macular holes in spite of surgery.^11^ Late reopening of previously repaired macular holes can occur secondarily to intraretinal and preretinal cellular remodeling, subsequent traction, progressive ERM formation or macular cystoid edema associated with cataract surgery.^15^ Spontaneous closure of late reopening macular holes after previous vitrectomy may be related to ERM contracture and bridging glial cell proliferation.^15^ Similar to our results, Distelmaier et al.^16^ demonstrated delayed macular hole closure after macular surgery consisting of PPV, ILM peeling, and gas (20% C2F6) tamponade. They reported a stage II idiopathic macular hole that spontaneously closed after 16 days of persistence. In contrast to Distelmaier et al.^16^ case, the persistence period following PPV was longer (5 months) in our patient. This inconsistency may be due to a larger preoperative hole diameter (671 microns) associated with more advanced macular hole stage. Unlike Distelmaier et al.^16^ study, the ILM could not be successfully removed in the our patient due to tight adhesions between the ILM and inner retina. In addition, postoperative OCT revealed an ERM in our case. We used the term “late spontaneous closure” instead of “delayed closure” because of the longer persistence period. In this case, a second surgery was planned including gas-fluid exchange with removal of the ILM and long-acting gas injection, but the patient wanted to postpone the second surgery. Monthly follow-up was done with OCT. Spontaneous closure or delayed healing started in the fifth month and continued through the eighth month after surgery. This delayed healing process can be explained by prolonged intraretinal and cellular remodeling with a mild ERM formation. The ILM remnants were suspected to be responsible for ERM formation.

In conclusion, despite the fact that possible mechanisms have not been elucidated yet, spontaneous macular hole closure may occur after a failed surgery. We suggest monitoring patients with OCT. However, the spontaneous closure of a persistent macular hole following PPV is quite a rare event, and early diagnosis and treatment of a persistent macular hole by additional vitreoretinal surgery must remain the primary goal.
